# Meta-Analysis of Apolipoprotein E Gene Polymorphism and Susceptibility of Myocardial Infarction

**DOI:** 10.1371/journal.pone.0104608

**Published:** 2014-08-11

**Authors:** Hong Xu, Haiqing Li, Jun Liu, Dan Zhu, Zhe Wang, Anqing Chen, Qiang Zhao

**Affiliations:** Department of Cardiac Surgery, Rui Jin Hospital, Shanghai Jiao Tong University School of Medicine, Shanghai, China; University of Milan, Italy

## Abstract

A number of case-control studies have been conducted to clarify the association between ApoE polymorphisms and myocardial infarction (MI); however, the results are inconsistent. This meta-analysis was performed to clarify this issue using all the available evidence. Searching in PubMed retrieved all eligible articles. A total of 33 studies were included in this meta-analysis, including 18752 MI cases and 18963 controls. The pooled analysis based on all included studies showed that the MI patients had a decreased frequency of the ε2 allele (OR = 0.78, 95% CI = 0.70–0.87) and an increased frequency of the ε4 allele (OR = 1.15, 95% CI = 1.10–1.20); The results also showed a decreased susceptibility of MI in the ε2ε3 *vs*. ε3ε3 analysis (OR = 0.79, 95% CI = 0.68–0.90) and in the ε2 *vs*. ε3 analysis (OR = 0.78, 95% CI = 0.69–0.89), an increased susceptibility of MI in the ε3ε4 vs. ε3ε3 analysis (OR = 1.26, 95% CI = 1.12–1.41), in the ε4 *vs*. ε3 analysis (OR = 1.22, 95% CI = 1.12–1.32) and in the ε4ε4 *vs*. ε3ε3 analysis (OR = 1.59, 95% CI = 1.15–2.19). However, there were no significant associations among polymorphisms and MI for the following genetic models: frequency of the ε3 allele (OR = 0.99, 95% CI = 0.96–1.02); ε2ε2 *vs*. ε3ε3 analysis (OR = 0.73, 95% CI = 0.40–1.32); or ε2ε4 *vs*. ε3ε3 analysis (OR = 1.10, 95% CI = 0.99–1.21). Our results suggested that the ε4 allele of ApoE is a risk factor for the development of MI and the ε2 allele of ApoE is a protective factor in the development of MI.

## Introduction

Myocardial infarction (MI) is a leading cause of death worldwide, and is a multifactorial disease, influenced by genetic and environmental factors [1]. The main risk factors for MI include hypertension, hypercholesterolemia, diabetes, obesity, and smoking. In addition, recent studies have also shown the importance of genetic factors caused by polymorphisms in the pathogenesis of MI [2]–[7].

Apolipoprotein E (Apo E) is a serum glycoprotein found in circulating chylomicrons (remnants), very low density lipoproteins, intermediate density lipoproteins and high-density lipoproteins [8]. ApoE is considered as an excellent candidate gene for studying the susceptibility to coronary heart disease (CHD) and MI because of its pivotal roles in the metabolisms of cholesterol and triglyceride [9]. The most extensively studied polymorphism in the ApoE gene codes for three variant alleles: ε2, ε3 and ε4, which yield six possible genotypes: ε2/ε2, ε2/ε3, ε2/ε4, ε3/ε3, ε3/ε4 and ε4/ε4 in general population [10]. The products of the three alleles differ in their properties such as their affinity for binding low density lipoprotein receptors and lipoprotein particles; therefore, this ApoE polymorphism could affect the serum levels of cholesterol and triglyceride, thus contributing to the progression of atherosclerosis. In fact, ApoE polymorphisms have been found to be associated with many lipid-related diseases and cardiovascular and cerebrovascular diseases [11]–[14].

Numerous studies have been conducted to explore the association of this ApoE polymorphism and CHD; some of the studies found a significant association between the ApoE ε4 allele and CHD [15]–[17]. A meta-analysis conducted in 2004 provided evidence that the ε4 allele of ApoE was a risk factor for the development of CHD [18]. Another meta- analysis conducted in 2013 further confirmed this finding in a Chinese population [19]. However, no meta-analysis has been conducted to explore the association between this ApoE gene polymorphism and MI. In spite of the presence of advanced CHD, only a subset of patients develops MI during their life. The reasons for these individual differences in susceptibility to MI are poorly understood. Therefore, it is important to explore the association between ApoE gene polymorphisms and MI. In fact, a number of case-control studies have been conducted to clarify the association between ApoE gene polymorphisms and MI [20]–[52]; however, the results are inconsistent. Therefore, we conducted this meta-analysis including all of the evidence produced to date to explore this issue.

## Materials and Methods

### Search strategy

We searched all published studies in the Pubmed database (up to January 20, 2014) using the following combination of keywords: “Apolipoprotein E” OR “ApoE” AND “acute coronary syndrome” OR “myocardial infarction” AND “polymorphism” OR “polymorphisms” OR “variants” OR “variant”. In addition, manual searches for related articles were also performed to avoid missing any relevant studies.

### Inclusion and exclusion criteria

The inclusion criteria for identified articles were as follows: 1) Case-control studies with full text articles on the relationship of ApoE polymorphisms and MI; 2) sufficient data for estimating an odds ratio (OR) with 95% confidence interval (CI). Those not designed as case-control studies, systemic reviews, those not written in English or Chinese, and those that provided no usable data, were excluded.

### Data extraction

Two authors independently extracted the data from all included studies using a predesigned data extraction table. The following information was extracted from each included article: first author, year of publication, ethnicity and country, source of controls, total numbers of MI cases and controls, distribution of genotypes and alleles in MI cases and controls, and evidence of conforming to the Hardy-Weinberg equilibrium (HWE).

### Statistical analysis

We firstly used chi-squared (χ^2^) test and and I^2^ statistic to assess heterogeneity across studies. A fixed effect model (Mantel–Haenszel) was used in the absence of heterogeneity. Otherwise, the random effect model (DerSimonian–Laird) was adopted. The strength of the association between the ApoE gene polymorphism and MI was assessed by odds ratios (ORs) with the corresponding 95% CI for each study. The ORs and their 95% CIs were assessed for the following seven genetic models: 1) ε2ε2 *vs*. ε3ε3; 2) ε2ε3 *vs*. ε3ε3; 3) ε2ε4 *vs*. ε3ε3; 4) ε3ε4 *vs*. ε3ε3; 5) ε4ε4 *vs*. ε3ε3; 6) ε2 vs. ε3; 7) ε4 vs. ε3. The allele frequencies of ε2, ε3 and ε4 were also assessed using the same method. Cumulative meta-analysis was also performed for the above genetic models. Subgroup analysis for ethnicity (Asian and Caucasian) was also performed. To find potential outliers, influence analysis was performed by omitting each study in turn. A funnel plot, calculated using Begg’s and Egger’s tests, was adopted for assessing potential publication bias. Statistical analysis was conducted using STATA statistical software (version 11; StataCorp, College Station, Texas, USA). A P value less than 0.05 was considered statistically significant.

## Results

### Literature selection and study characteristics

**Figure 1:**
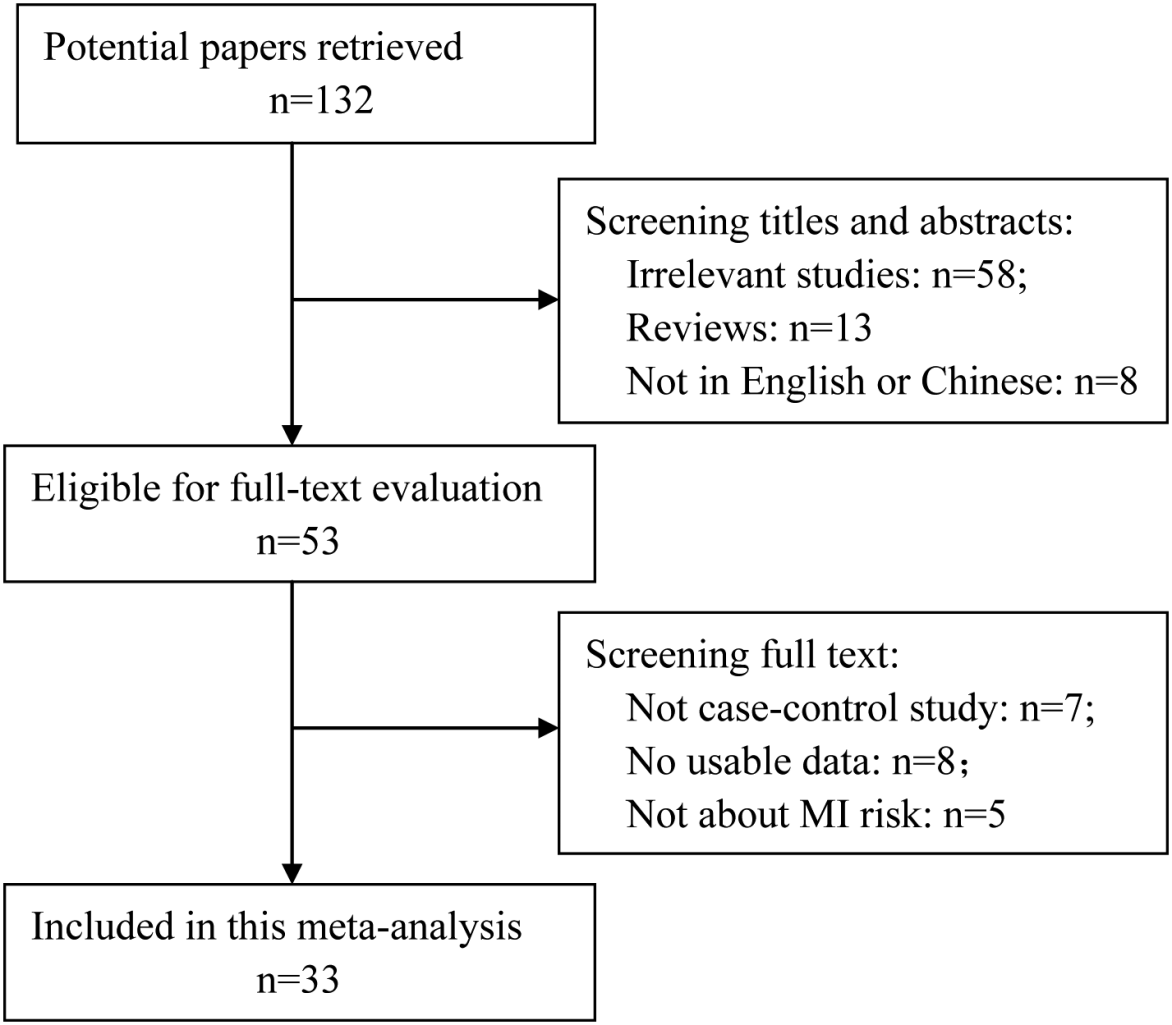
Flowchart of the study selection.

One hundred and thirty two articles were retrieved from PubMed, 79 of which were excluded after screening the titles and abstracts (58 were irrelevant studies, 13 were reviews and eight were not published in English or Chinese). Fifty-three articles were selected for detailed assessment, which excluded a further 20 articles (seven were not case-control studies, eight had no usable data (no case and control numbers according to the genotypes) and five were not about MI). Finally, 33 studies were included in this meta-analysis, which included 18752 MI cases and 18963 controls. The detailed selection procedure is shown in **Figure 1**. There were three studies did not follow the HWE. The detailed characteristics of the included studies are shown in **Table 1**. The present study met the PRISMA statement requirements (**Checklist S1** and **Figure 1**).

**Table 1:** Detailed characteristics of studies included in this meta-analysis.

							Genotypes distribution (Cases/controls)								
Study [Reference]	Year	Country	Ethnicity	Study	HWE	Total sample	ε2/ε2	ε2/ε3	ε3/ε3	ε2/ε4	ε3/ε4	ε4/ε4	ε2	ε3	ε4
				type		Case/Control									
Utermann1984[20]	1984	Germany	Caucasion	HCC	Yes	523/1031	7/120	x68/124	333/617	11/15	92/236	12/29	86/359	493/977	115/280
Cumming1984[21]	1984	Scotland	Caucasion	PCC	Yes	239/400	0/2	18/51	128/233	10/11	77/99	6/4	28/64	223/383	93/114
Lenzen1986[22]	1986	France	Caucasion	PCC	Yes	570/624	1/6	50/67	360/393	10/20	137/125	12/13	61/93	547/585	159/158
Eichner 1993[23]	1993	USA	Caucasion	PCC	Yes	114/412	0/2	16/35	67/276	0/4	30/85	1/10	16/41	113/396	31/99
Luc 1994[24]	1994	France	Caucasion	PCC	Yes	574/680	3/6	54/92	352/428	14/14	133/126	18/14	71/112	539/646	165/154
Hergenc 1995'[25]	1995	Turkey	Caucasion	HCC	Yes	50/60	0/0	7/6	41/47	0/2	2/5	0/0	7/8	50/58	2/7
Kim 1995[26]	1995	Korea	Asian	HCC	Yes	97/137	2/1	17/25	57/95	0/4	20/12	1/0	19/30	94/132	21/16
Nakai 1998[27]	1998	Japan	Asian	PCC	Yes	254/422	0/0	10/16	178/327	2/4	52/74	6/1	12/20	240/417	60/79
Scaglione1999[28]	1999	Italy	Caucasion	HCC	No	98/98	NR	NR	NR	NR	NR	NR	3/3	84/87	11/8
Lambert 2000[29]	2000	France	Caucasion	PCC	Yes	567/678	3/4	67/100	332/420	0/3	152/138	18/13	70/107	551/658	170/154
Benes2000[30]	2000	Czech	Caucasion	PCC	Yes	114/222	1/0	12/30	71/147	3/2	23/43	4/0	16/32	106/220	30/45
Batalla 2000[31]	2000	Spain	Caucasion	PCC	Yes	220/200	0/0	9/18	174/151	1/1	32/28	4/2	10/19	215/197	37/31
Raslová 2001[32]	2001	Canada	Caucasion	PCC	Yes	69/69	2/1	8/5	46/47	1/0	11/15	1/1	11/6	65/67	13/16
Bai 2001[33]	2001	China	Asian	PCC	Yes	47/50	0/0	4/5	40/39	0/0	6/3	0/0	4/5	50/47	6/3
Freitas 2002[34]	2002	Australia	Caucasion	PCC	Yes	411/624	3/4	24/67	254/372	9/15	111/147	10/19	36/86	389/586	130/181
Mamotte 2002[35]	2002	Australia	Caucasion	PCC	Yes	359/639	4/4	24/68	217/383	7/16	96/149	11/19	35/88	337/600	114/184
Kolovou 2002[36]	2002	Greece	Caucasion	PCC	Yes	124/240	0/0	3/34	94/159	0/5	27/40	0/2	3/39	124/233	27/47
Keavney 2003[37]	2003	UK	Caucasion	PCC	Yes	4487/5757	NR	440/686	2566/3384	NR	1206/1376	NR	440/686	4212/5446	1206/1376
Kolovou 2003[38]	2003	Greece	Caucasion	PCC	Yes	165/165	0/0	3/16	129/118	1/4	29/23	1/0	4/20	161/157	31/27
Kumar 2003[39]	2003	India	Caucasion	PCC	Yes	35/45	0/2	6/9	12/32	1/0	6/0	10/2	7/9	24/41	17/2
Marques 2003[40]	2003	France	Caucasion	HCC	Yes	400/338	NR	NR	272/228	NR	NR	NR	37/40	272/228	91/70
Keavney 2004[41]	2004	UK	Caucasion	PCC	Yes	4685/3460	NR	440/406	2566/1949	1206/810	NR	NR	1646/1216	3006/2355	1206/810
Ranjith 2004[42]	2004	South Africa	Caucasion	PCC	Yes	195/300	0/3	7/18	139/228	3/3	45/43	1/5	10/24	191/289	49/51
Baum 2006[43]	2006	China	Asian	PCC	Yes	231/331	0/2	13/60	164/203	4/6	46/39	4/1	17/68	223/302	54/46
Aasvee 2006[44]	2006	Estonia	Caucasion	PCC	Yes	71/85	1/1	4/13	45/52	2/3	16/16	3/0	7/17	65/81	21/19
Koch 2008[45]	2008	Germany	Caucasion	PCC	Yes	3657/1211	26/7	402/164	2279/736	63/23	809/263	78/18	491/194	3490/1163	950/304
Kolovou 2009[46]	2009	Greece	Caucasion	PCC	Yes	124/240	NR	NR	NR	NR	NR	NR	5/19	106/197	13/24
Bahri 2008[47]	2008	Tunisia	Caucasion	PCC	Yes	80/100	0/0	6/8	61/78	0/1	13/13	0/0	6/9	80/199	13/14
Martinelli 2009[48]	2009	Italy	Caucasion	HCC	Yes	394/287	NR	NR	NR	NR	NR	NR	34/25	285/220	76/42
Al-Bustan 2009[49]	2009	Kuwaiti	Caucasion	HCC	No	88/122	4/9	2/2	72/98	2/3	8/9	0/1	6/11	90/33	16/5
Onrat 2012[50]	2012	Turkey	Caucasion	PCC	Yes	36/100	0/0	12/4	72/27	0/0	16/4	0/1	12/4	100/35	16/5
Tanguturi 2013[51]	2013	USA	Caucasion	HCC	Yes	202/210	0/0	8/14	142/167	4/3	37/23	11/3	12/17	187/204	52/29
Zende 2013[52]	2013	India	Caucasion	HCC	No	150/150	6/7	13/16	59/85	7/4	22/14	43/24	26/27	94/115	72/42

HWE, Hardy-Weinberg equilibrium; NR, not reported; Cases, MI patients; HCC, hospital based case-control study; PCC, population based case-control study.

### Quantitative data synthesis

**Figure 2:**
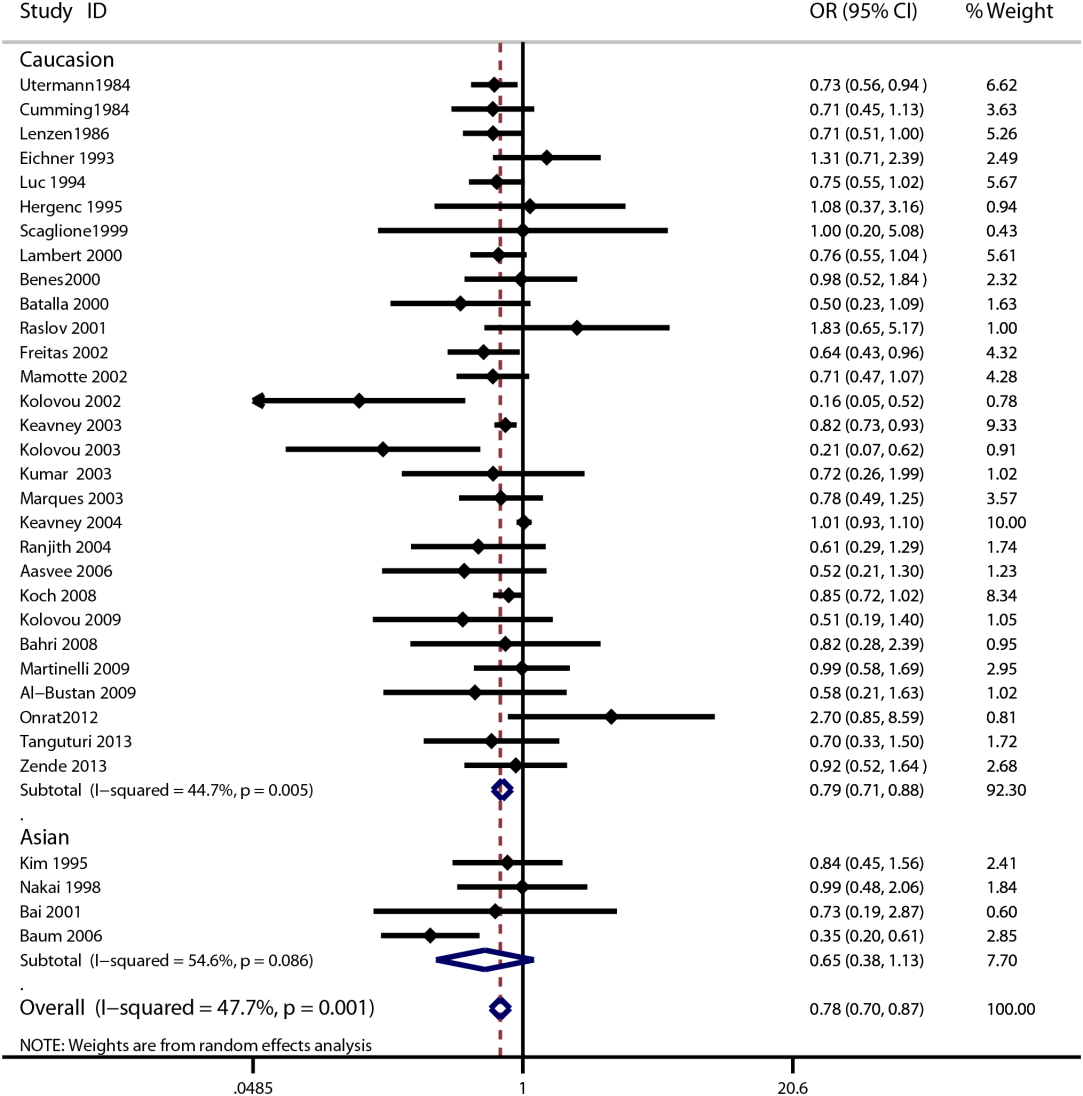
Forest plot for ApoE gene polymorphism and MI risk in the ε2 allele frequency analysis.

**Figure 3:**
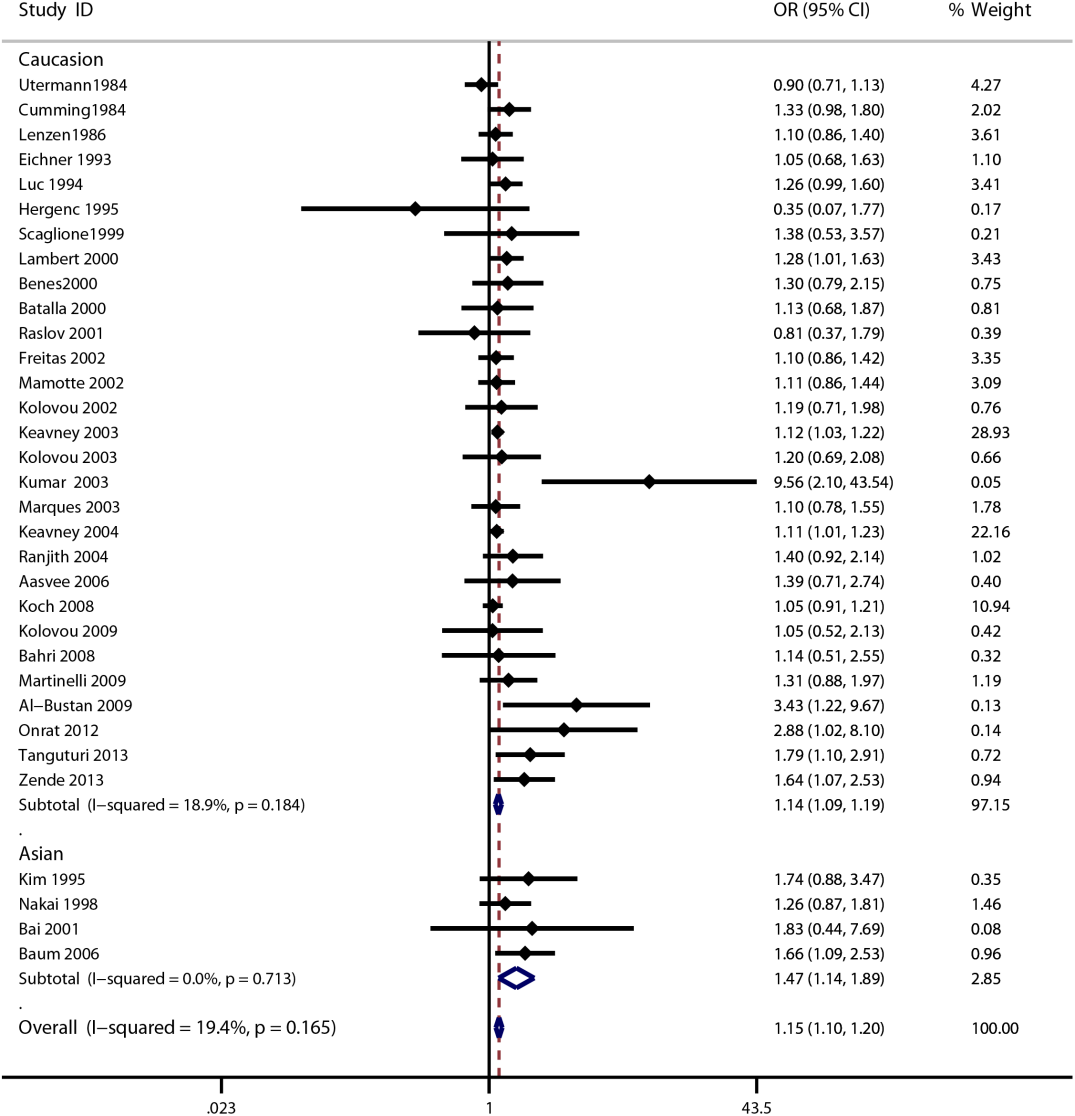
Forest plot for ApoE gene polymorphism and MI risk in the ε4 allele frequency analysis.

**Figure 4:**
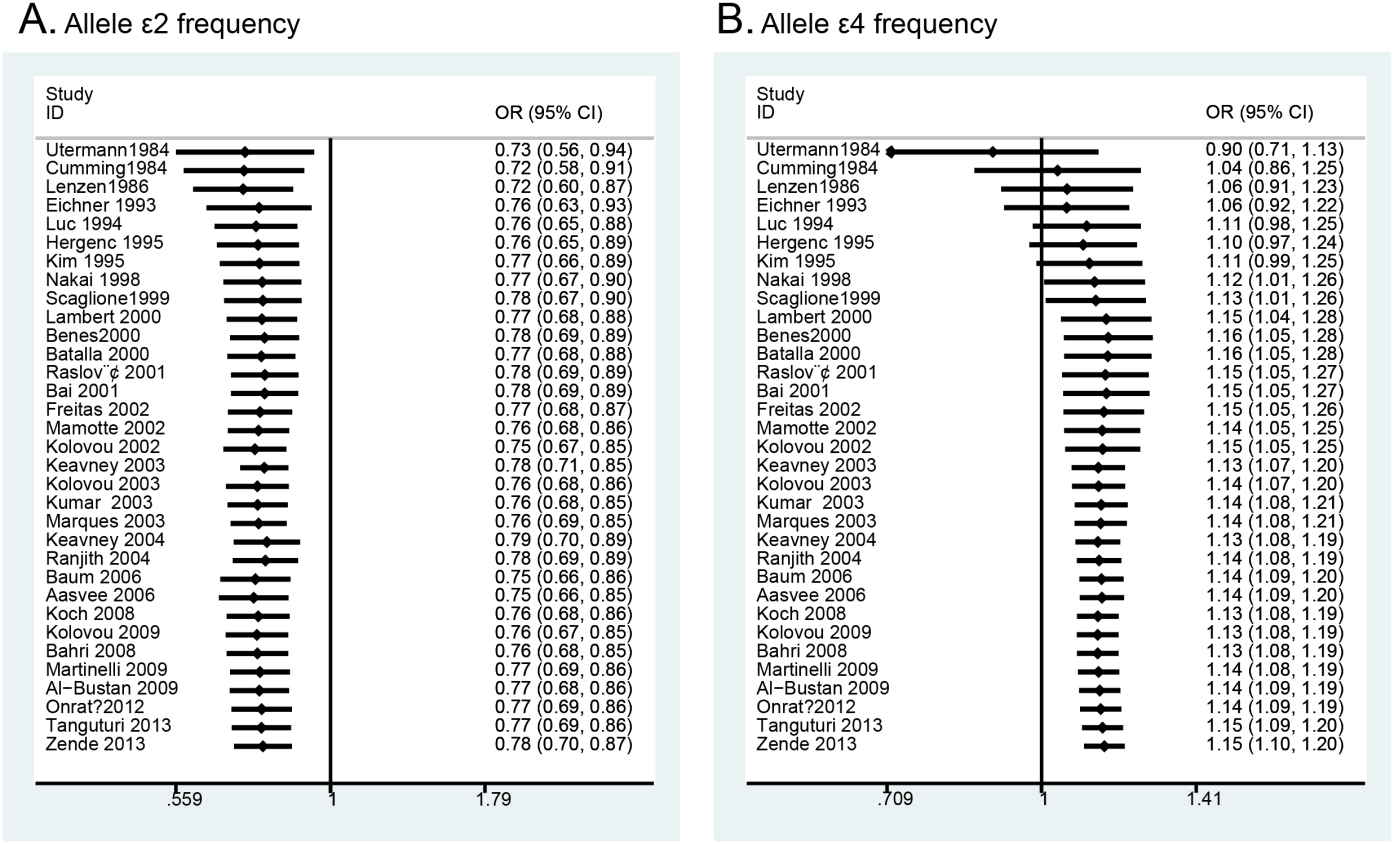
Cumulative meta-analysis of ApoE gene polymorphism and MI risk: A) ε2 allele frequency analysis; B) ε4 allele frequency analysis.

The meta-analysis of the included studies showed that there was significant association between the ApoE gene polymorphism and MI. The results showed that the MI patients had a decreased frequency of the ε2 allele (OR = 0.78, 95% CI = 0.70–0.87, **Figure 2**) and an increased frequency of the ε4 allele (OR = 1.15, 95% CI = 1.10–1.20, **Figure 3**). The results also showed a decreased susceptibility of MI in the ε2ε3 *vs*. ε3ε3 analysis (OR = 0.79, 95% CI = 0.68–0.90, **Figure S1**), and in the ε2 *vs*. ε3 analysis (OR = 0.78, 95% CI = 0.69–0.89, **Figure S4**), and an increased susceptibility of MI in the ε3ε4 *vs*. ε3ε3 analysis (OR = 1.26, 95% CI = 1.12–1.41, **Figure S2**) in the ε4ε4 *vs*. ε3ε3 analysis (OR = 1.59, 95% CI = 1.15–2.19, **Figure S3**) and in the ε4 *vs*. ε3 analysis (OR = 1.22, 95% CI = 1.12–1.32, **Figure S5**). However, there were no significant associations among polymorphisms and MI for the following genetic models: frequency of ε3 allele (OR = 0.99, 95% CI = 0.96–1.02); ε2ε2 *vs*. ε3ε3 analysis (OR = 0.73, 95% CI = 0.40–1.32); ε2ε4 *vs*. ε3ε3 analysis (OR = 1.10, 95% CI = 0.99–1.21). The detailed results are shown in **Table 2**. Cumulative analysis further confirmed the results (**Figure 4** and **Figure S6**).

**Table 2:** Results of meta-analysis of ApoE polymorphism and MI.

	Overall		Caucasion		Asian	
Analysis	OR (95% CI)	P/P_het_	OR (95% CI)	P/P_het_	OR (95% CI)	P/P_het_
**ε2ε2 vs. ε3ε3**	0.73 (0.40–1.32)	0.29/0.005	0.70 (0.38–1.31)	0.27/0.004	1.07 (0.08–13.78)	0.96/0.18
**ε2ε3 vs. ε3ε3**	0.79 (0.68–0.90)	0.001/0.001	0.80 (0.70–0.92)	0.001/0.008	0.70 (0.31–1.60)	0.84/0.007
**ε2ε4 vs. ε3ε3**	1.10 (0.99–1.21)	0.07/0.70	1.10 (1.00–1.21)	0.05/0.63	0.66 (0.26–1.70)	0.39/0.61
**ε3ε4 vs. ε3ε3**	1.26 (1.12–1.41)	<0.001/0.001	1.23 (1.09–1.38)	0.001/0.001	1.51 (1.14–2.00)	0.004/0.39
**ε4ε4 vs. ε3ε3**	1.59 (1.15–2.19)	0.005/0.04	1.47 (1.07–2.02)	0.02/0.05	6.95 (1.75–27.65)	0.006/0.85
**ε2 vs. ε3**	0.78 (0.69–0.89)	<0.001/0.04	0.80 (0.71–0.90)	<0.001/0.04	0.67 (0.37–1.23)	0.20/0.22
**ε4 vs. ε3**	1.22 (1.12–1.32)	<0.001/0.02	1.20 (1.10–1.30)	<0.001/0.02	1.49 (1.15–1.93)	0.002/<0.001
**ε2 allele** **frequency**	0.78 (0.70–0.87)	<0.001/0.001	0.79 (0.71–0.88)	<0.001/0.005	0.65 (0.38–1.13)	0.13/0.09
**ε3 allele** **frequency**	0.99 (0.96–1.02)	0.38/1.00	0.99 (0.96–1.02)	0.39/1.00	0.99 (0.86–1.13)	0.22/0.94
**ε4 allele** **frequency**	1.15 (1.10–1.20)	0.001/0.17	1.14 (1.09–1.19)	0.001/0.18	1.47 (1.14–1.89)	0.003/0.70

P, p value of the test on the association estimate; Phet, p value of the heterogeneity test.

### Tests of heterogeneity and subgroup analysis

Significant between-study heterogeneity existed in the analyses of seven genetic models: ε2 ε2 *vs*. ε3ε3 (p = 0.005); ε2ε3 *vs*. ε3ε3 (p = 0.001); ε3ε4 *vs*. ε3ε3 (p = 0.001); ε4ε4 *vs*. ε3ε3 (p = 0.04), ε2 *vs*. ε3 (p = 0.04), ε2 *vs*. ε3 (p = 0.02) and the ε2 allele frequency (p = 0.001). A random effects model was adopted for these analyses.

Furthermore, we performed subgroup analysis based on ethnicity and found a decreased susceptibility of MI in the ε2ε3 *vs*. ε3ε3 analysis (OR = 0.80, 95% CI = 0.70–0.92) and ε2 allele frequency (OR = 0.79, 95% CI = 0.71–0.88) among Caucasian populations. We also found an increased susceptibility of MI in the ε3ε4 *vs*. ε3ε3 analysis (OR = 1.23, 95% CI = 1.09–1.38), ε4ε4 *vs*. ε3ε3 analysis (OR = 1.47, 95% CI = 1.07–2.02) and the ε4 allele frequency (OR = 1.14, 95% CI = 1.09–1.19) among Caucasian populations. Among Asian populations, we also found an increased susceptibility of MI in the ε3ε4 *vs*. ε3ε3 analysis, ε4ε4 *vs*. ε3ε3 analysis and for the ε4 allele frequency; the detailed results are shown in **Table 2**.

### Sensitivity analysis

**Figure 5:**
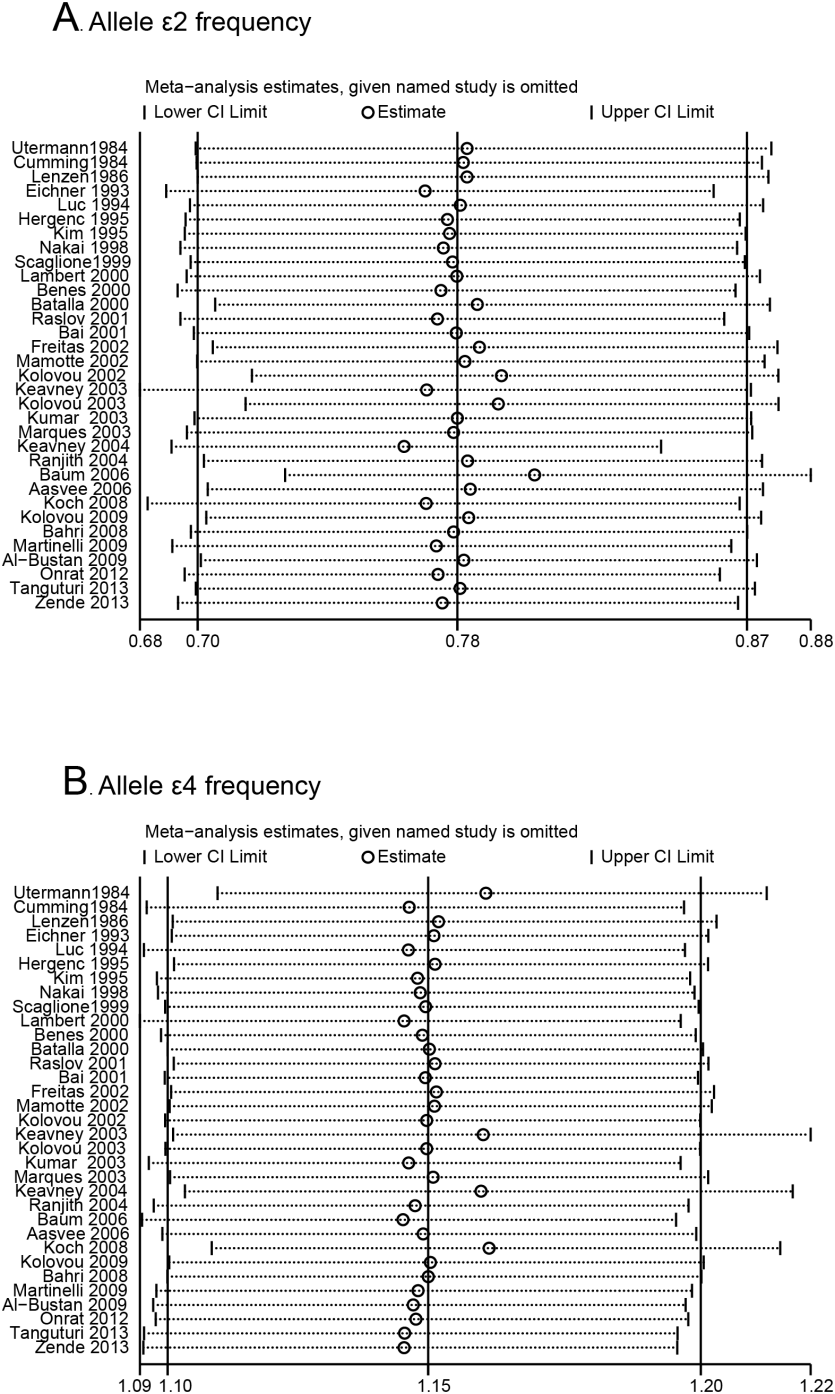
Influence analysis of ApoE gene polymorphism and MI risk: A) ε2 allele frequency analysis; B) ε4 allele frequency analysis.

We conducted influence analysis to assess the sensitivity of each individual study on the pooled ORs by sequential omission of each individual study. The results suggested that no individual study significantly affected the pooled ORs in the ε2 allele and ε4 allele frequency analysis (**Figure 5**), and in the ε2ε3 *vs*. ε3ε3 analysis, ε3ε4 *vs*. ε3ε3 analysis and ε4ε4 *vs*. ε3ε3 analysis (**Figure S7**).

### Publication bias

**Figure 6:**
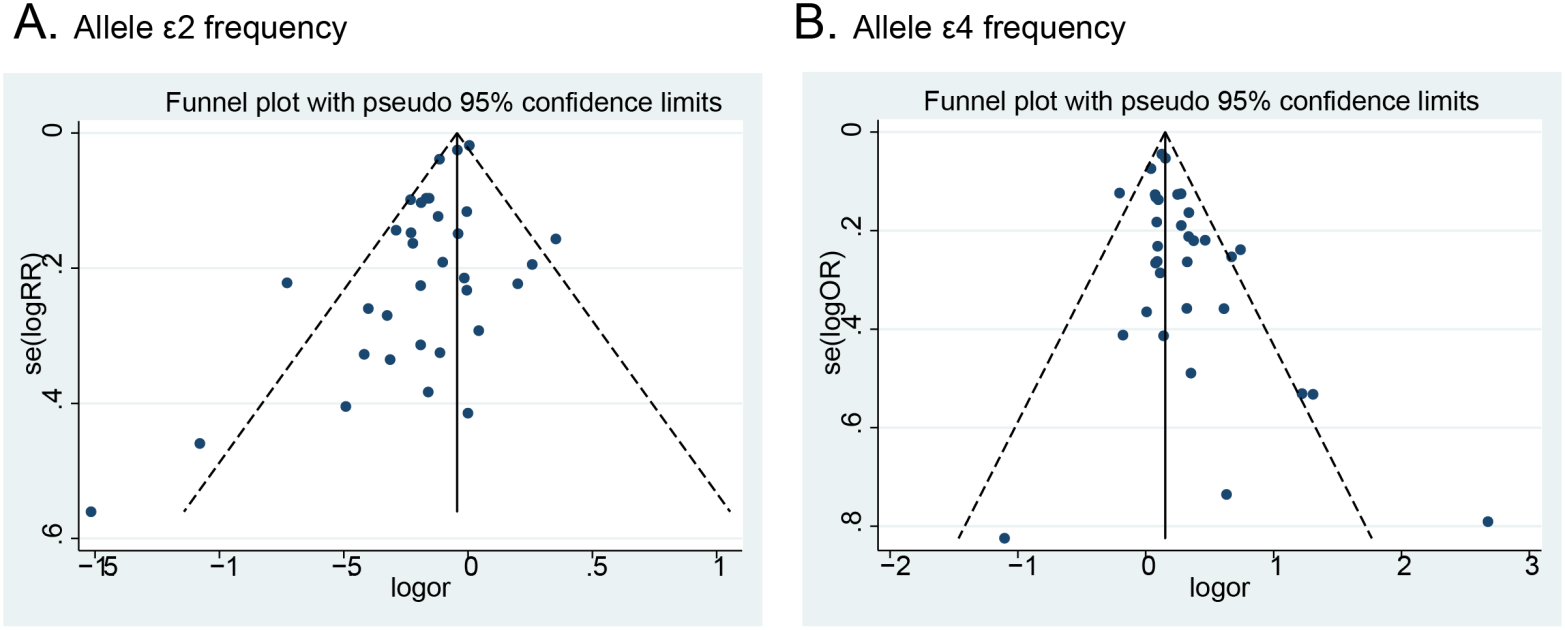
Funnel plot of ApoE gene polymorphism and MI risk: A) ε2 allele frequency analysis; B) ε4 allele frequency analysis.

Funnel plots examined potential publication bias qualitatively and no obvious asymmetry was observed in any genetic model, as shown in **Figure 6**. Furthermore, the results from Begg’s and Egger’s tests did not provide any evidence of publication bias (**Table S1**).

## Discussion

To the best of our knowledge, this is the first meta-analysis to evaluate the association between an ApoE polymorphism and susceptibility of MI. In this meta-analysis, we discovered an increased susceptibility of MI in the ε4 allele frequency analysis. Moreover, the individuals with ε2ε4 genotype, ε3ε4 genotype and ε4ε4 genotype had a significantly higher susceptibility of developing MI compared to those with the ε3ε3 genotype. Therefore, it is reasonable to assume that the ε4 allele of ApoE is an risk factor for the development of MI. These results were consistent with a previous meta-analysis, which showed that ε4 allele of ApoE is a risk factor for the development of CHD [11], [12]. In addition, we found a decreased susceptibility of MI in the ε2 allele frequency analysis and in the ε2ε3 vs. ε3ε3 analysis, which indicate that the ε2 allele is a protective factor in the development of MI. Cumulative meta-analysis also confirmed these findings. Considering the large sample size in the pooled analysis in this meta-analysis, we believe that our results are robust and reliable.

ApoE is a multifunctional protein that plays an important role in the metabolism of cholesterol and triglycerides, by binding to its receptors to help mediate clearance of chylomicron and remnant particles [53]. The three common isoforms, ε2, ε3 and ε4, have different receptor-binding abilities and could yield different circulating levels of cholesterol and triglycerides. Compared with ε3 homozygotes, carriers of the ε2 allele have lower circulating cholesterol levels, whereas carriers of the ε4 allele appear to have higher plasma levels of total and low-density lipoprotein cholesterol [54]. According to these mechanisms, our meta-analysis suggested that carrying the ε4 allele is a risk factor for MI and that the ε2 allele has a protective role in the development of MI. When stratifying the studies by ethnicity, the ε4 allele remained a risk factor and the ε2 allele was still protective in the development of MI among Caucasian populations; however, only the ε4 allele remained as a risk factor for MI among Asian population. This may be due to the small sample size in the analysis among Asian populations; in fact, there were only four studies that included Asian populations [19], [20], [26], [36]. Therefore, further studies are warranted among Asian populations. In addition, genotype distributions in the controls from Scaglione’s study [28], Bustan’s study [49] and Zende’s study [52] were not in agreement with HWE, therefore, the results may be biased. However, sensitivity analysis suggested that the pooled results were not significantly changed after excluding the three studies (data not shown). This may be due to the large sample size even though the three studies were excluded.

Although the primary results of this meta-analysis are suggestive, some limitations still exist. First, between-study heterogeneity existed in some of the genetic model analysis, which may have affected the results of the present meta-analysis, although a random effects model was adopted for these analyses. Second, publication bias may have occurred because our analyses were based wholly on published studies only in English and Chinese. Third, the results of this meta-analysis were based on unadjusted estimates because of the lack of adjusted estimates. Currently, some risk factors have been identified for MI, such as hypertension, hypercholesterolemia, diabetes, obesity and smoking. A more precise analysis should be performed if these data could be extracted from primary articles.

In conclusion, this comprehensive meta-analysis has evaluated all published data currently available on the association between the ApoE polymorphism and MI. Our meta-analysis suggested that the ε4 allele of ApoE is an risk factor for the development of MI and the ε2 allele of ApoE is a protective factor in the development of MI. This may be explained by the fact that ε4 allele of ApoE elevates the plasma levels of total and low-density lipoprotein cholesterol while the ε2 allele of ApoE lowers the circulating cholesterol levels. Further studies with larger sample sizes are warranted among Asian populations.

## Supporting Information

Figure S1 Forest plot for ApoE gene polymorphism and MI risk in the genetic model of ε2ε3 vs. ε3ε3 analysis.
(TIF)

Figure S2 Forest plot for ApoE gene polymorphism and MI risk in the genetic model of ε3ε4 vs. ε3ε3 analysis.
(TIF)

Figure S3 Forest plot for ApoE gene polymorphism and MI risk in the genetic model of ε4ε4 vs. ε3ε3 analysis.
(TIF)

Figure S4 Forest plot for ApoE gene polymorphism and MI risk in the genetic model of ε2 vs. ε3 analysis.
(TIF)

Figure S5 Forest plot for ApoE gene polymorphism and MI risk in the genetic model of ε4 vs. ε3 analysis.
(TIF)

Figure S6 Cumulative meta-analysis of ApoE gene polymorphism and MI risk: A) ε2ε3 vs. ε3ε3 analysis; B) ε3ε4 vs. ε3ε3 analysi; C) ε4ε4 vs. ε3ε3 analysis.
(TIF)

Figure S7 Influence analysis of ApoE gene polymorphism and MI risk: A) ε2ε3 vs. ε3ε3 analysis; B) ε3ε4 vs. ε3ε3 analysi; C) ε4ε4 vs. ε3ε3 analysis.
(TIF)

Table S1 Results of Egger’s and Begger’s test.
(XLS)

Checklist S1 PRISMA Checklist.
(DOC)
