# Supplementary figures and images for: Meta-Analysis of Apolipoprotein E Gene Polymorphism and Susceptibility of Myocardial Infarction
Source: PLoS One. 2014 Aug 11;9(8):e104608. doi: 10.1371/journal.pone.0104608 (PMC4128680; doi:10.1371/journal.pone.0104608)

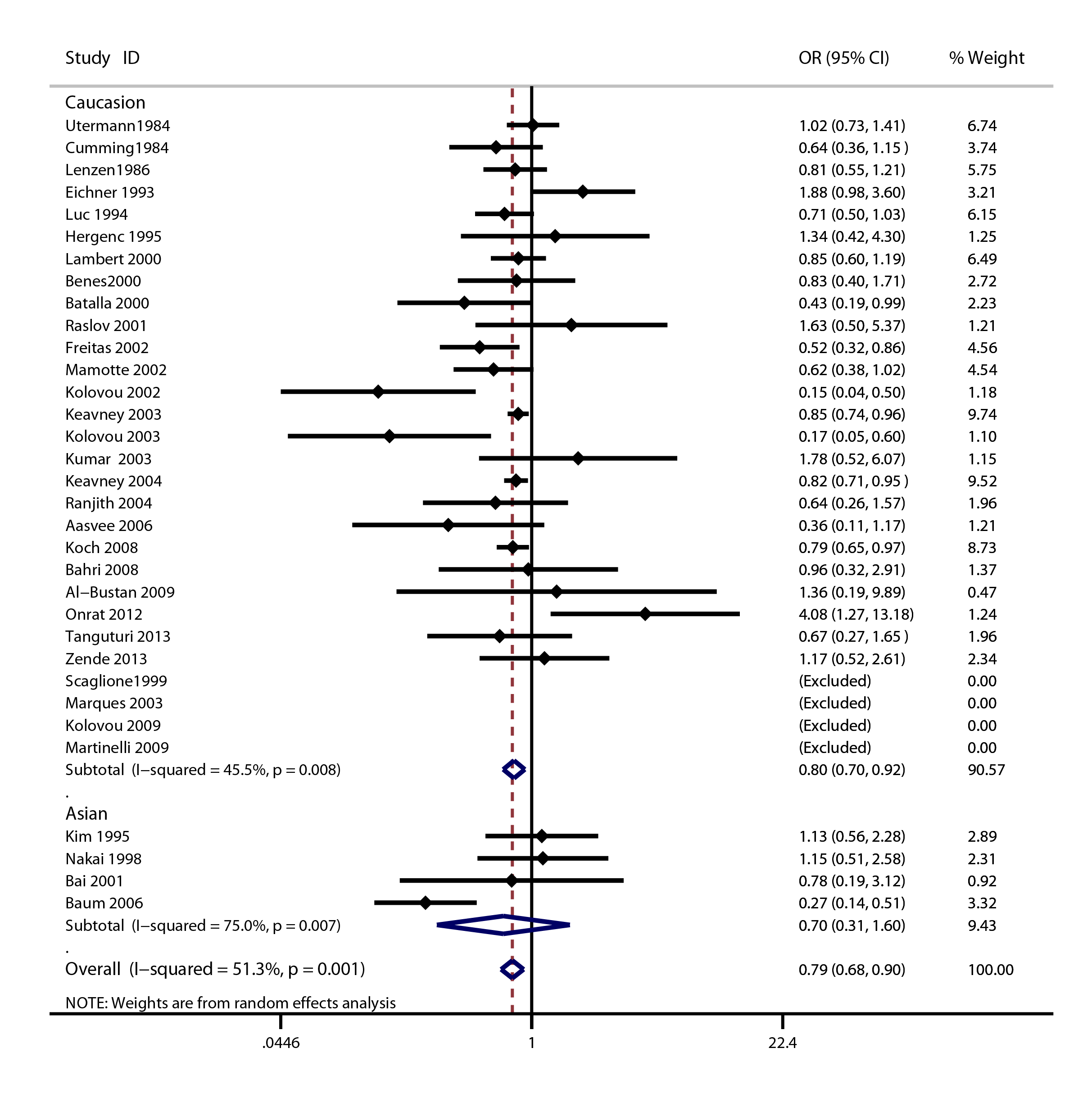

Supplement: Figure S1 — Forest plot for ApoE gene polymorphism and MI risk in the genetic model of ε2ε3 vs. ε3ε3 analysis. (TIF) [file pone.0104608.s001.tif]

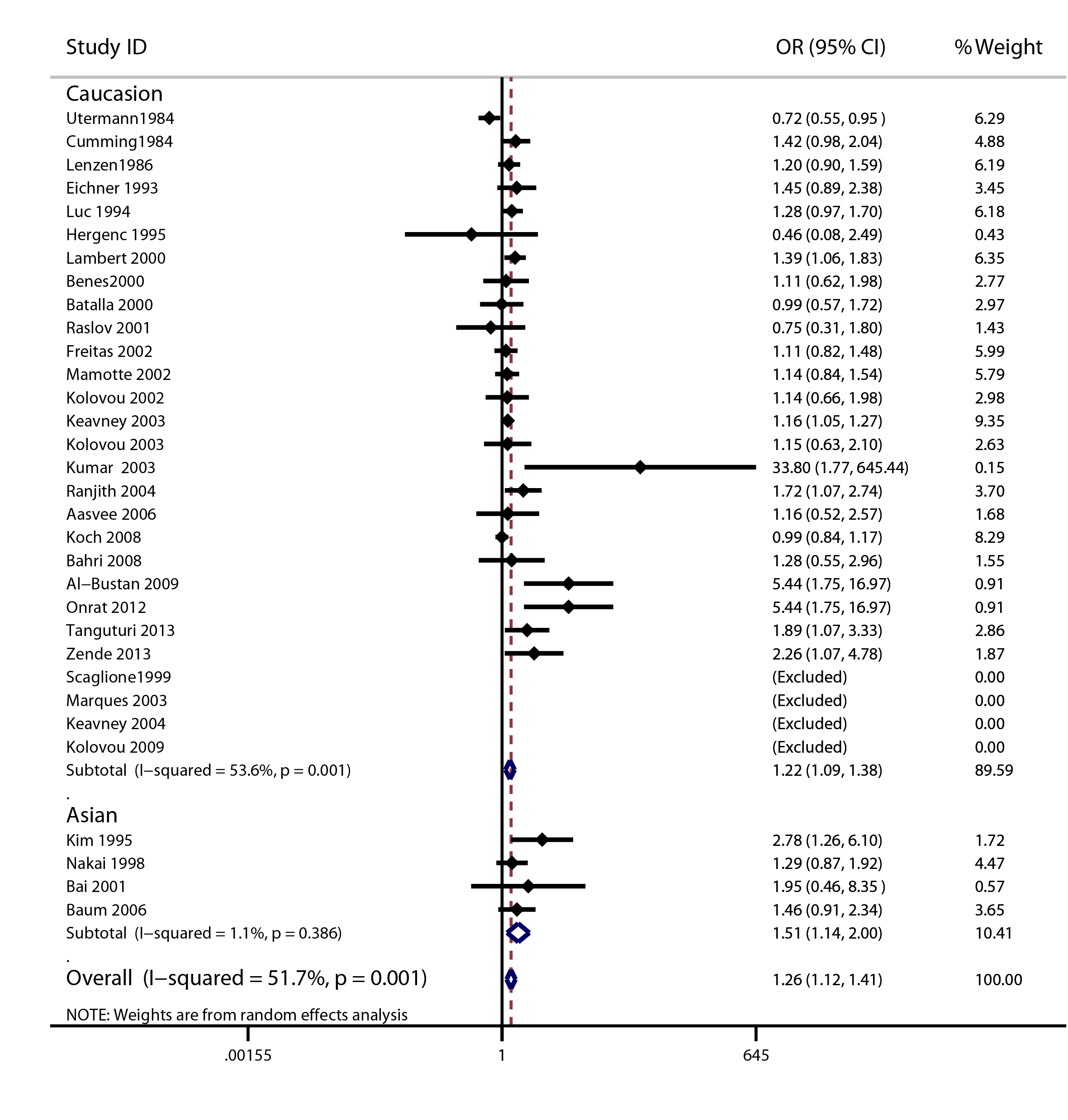

Supplement: Figure S2 — Forest plot for ApoE gene polymorphism and MI risk in the genetic model of ε3ε4 vs. ε3ε3 analysis. (TIF) [file pone.0104608.s002.tif]

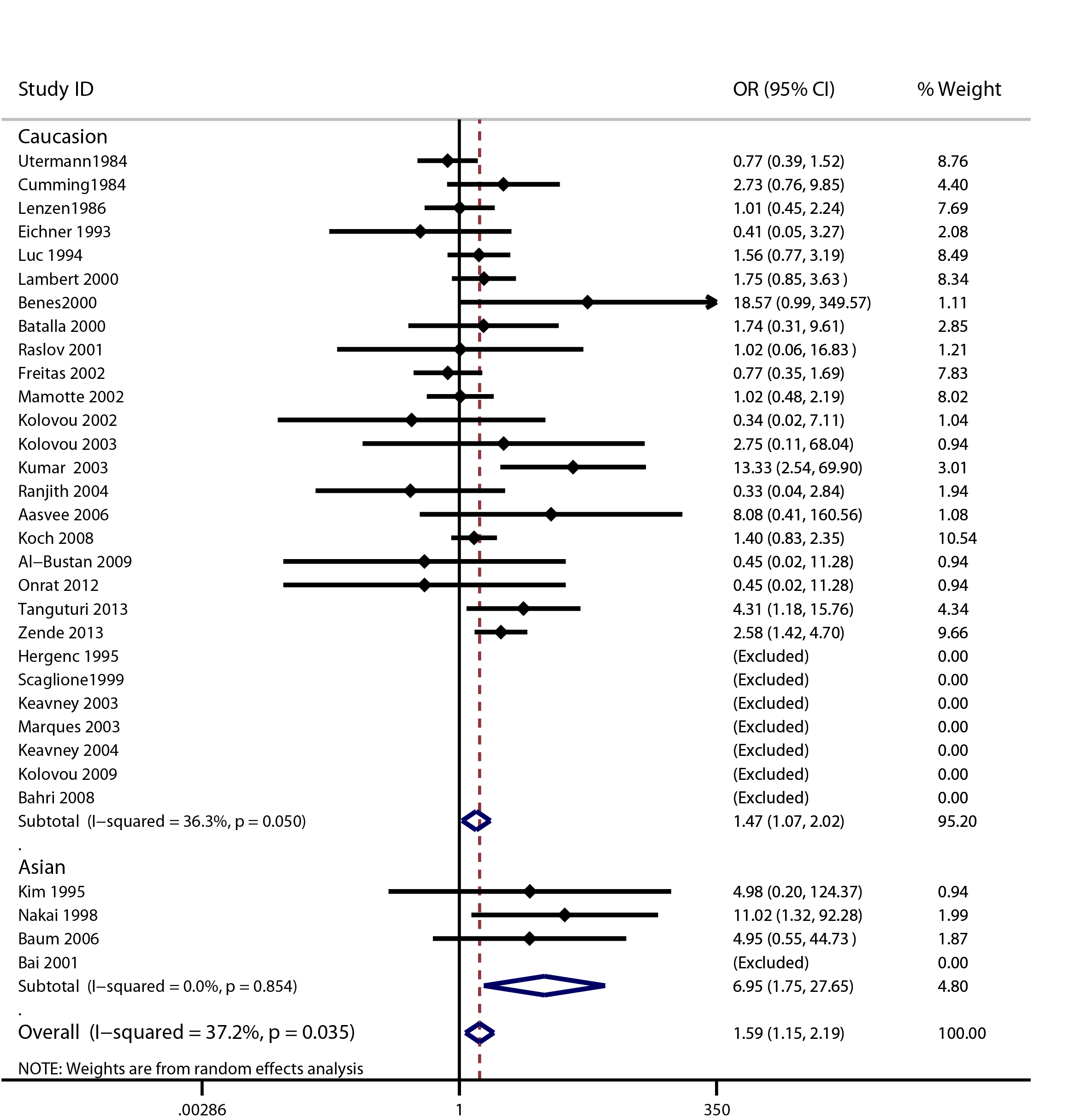

Supplement: Figure S3 — Forest plot for ApoE gene polymorphism and MI risk in the genetic model of ε4ε4 vs. ε3ε3 analysis. (TIF) [file pone.0104608.s003.tif]

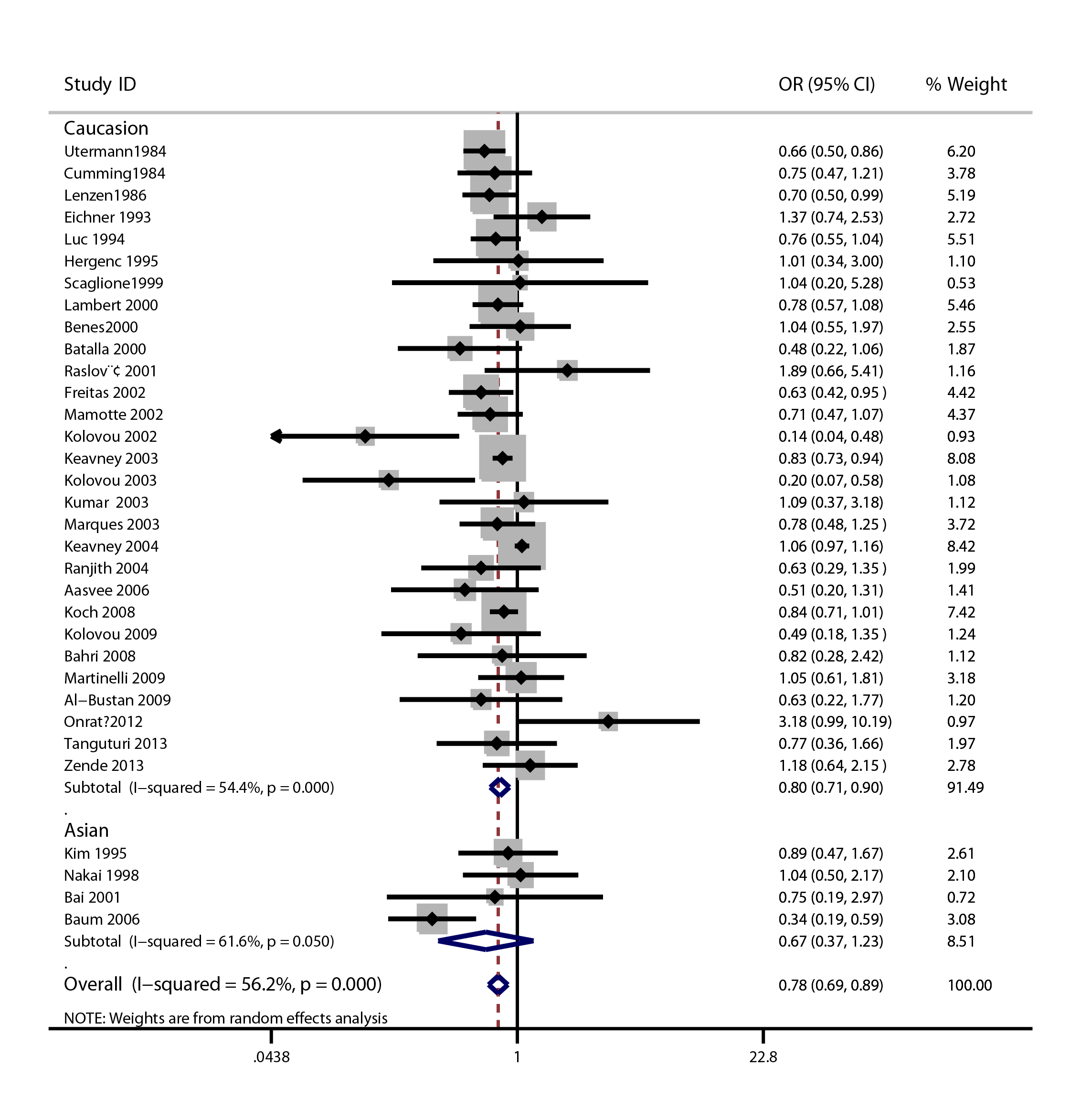

Supplement: Figure S4 — Forest plot for ApoE gene polymorphism and MI risk in the genetic model of ε2 vs. ε3 analysis. (TIF) [file pone.0104608.s004.tif]

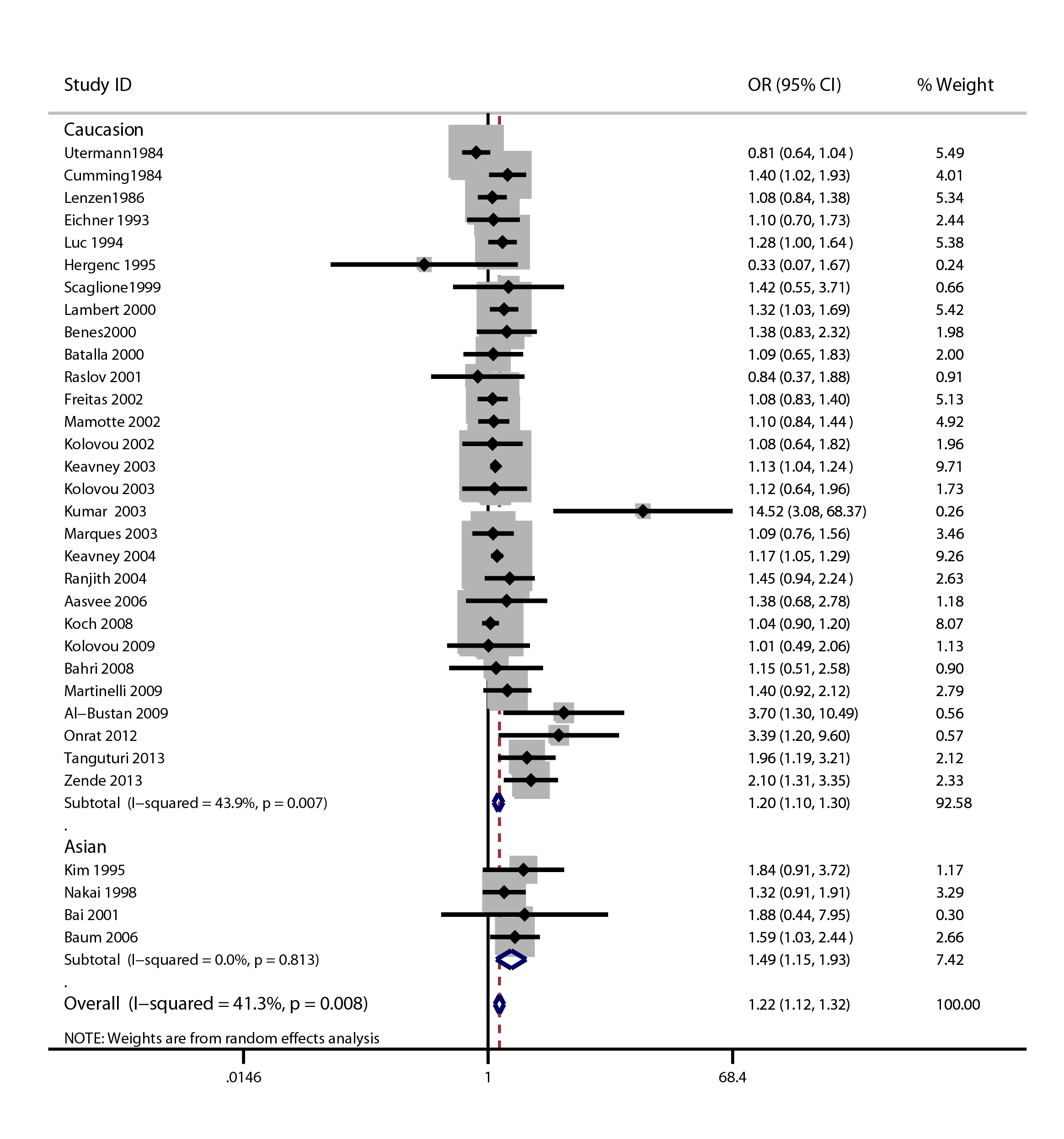

Supplement: Figure S5 — Forest plot for ApoE gene polymorphism and MI risk in the genetic model of ε4 vs. ε3 analysis. (TIF) [file pone.0104608.s005.tif]

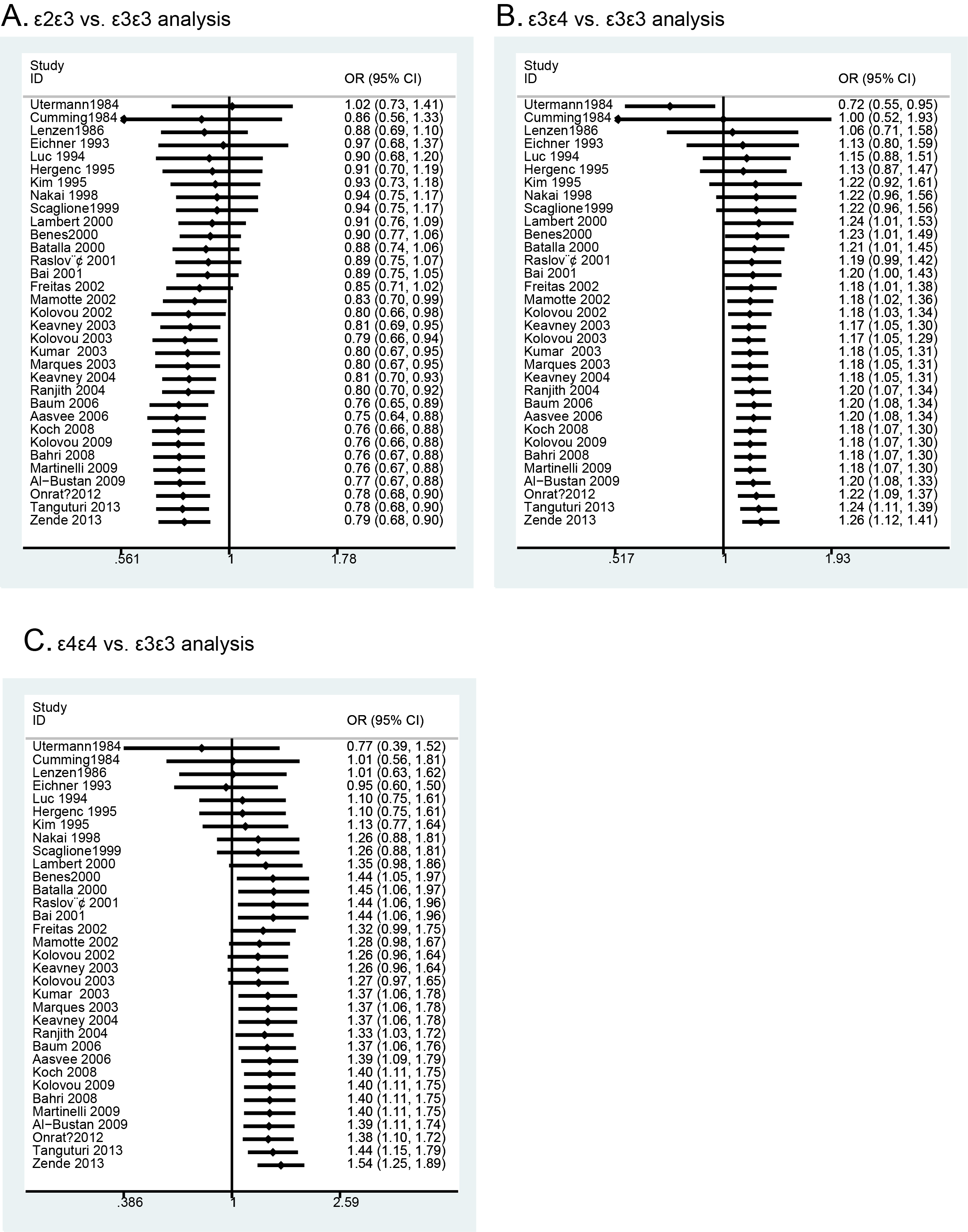

Supplement: Figure S6 — Cumulative meta-analysis of ApoE gene polymorphism and MI risk: A) ε2ε3 vs. ε3ε3 analysis; B) ε3ε4 vs. ε3ε3 analysi; C) ε4ε4 vs. ε3ε3 analysis. (TIF) [file pone.0104608.s006.tif]

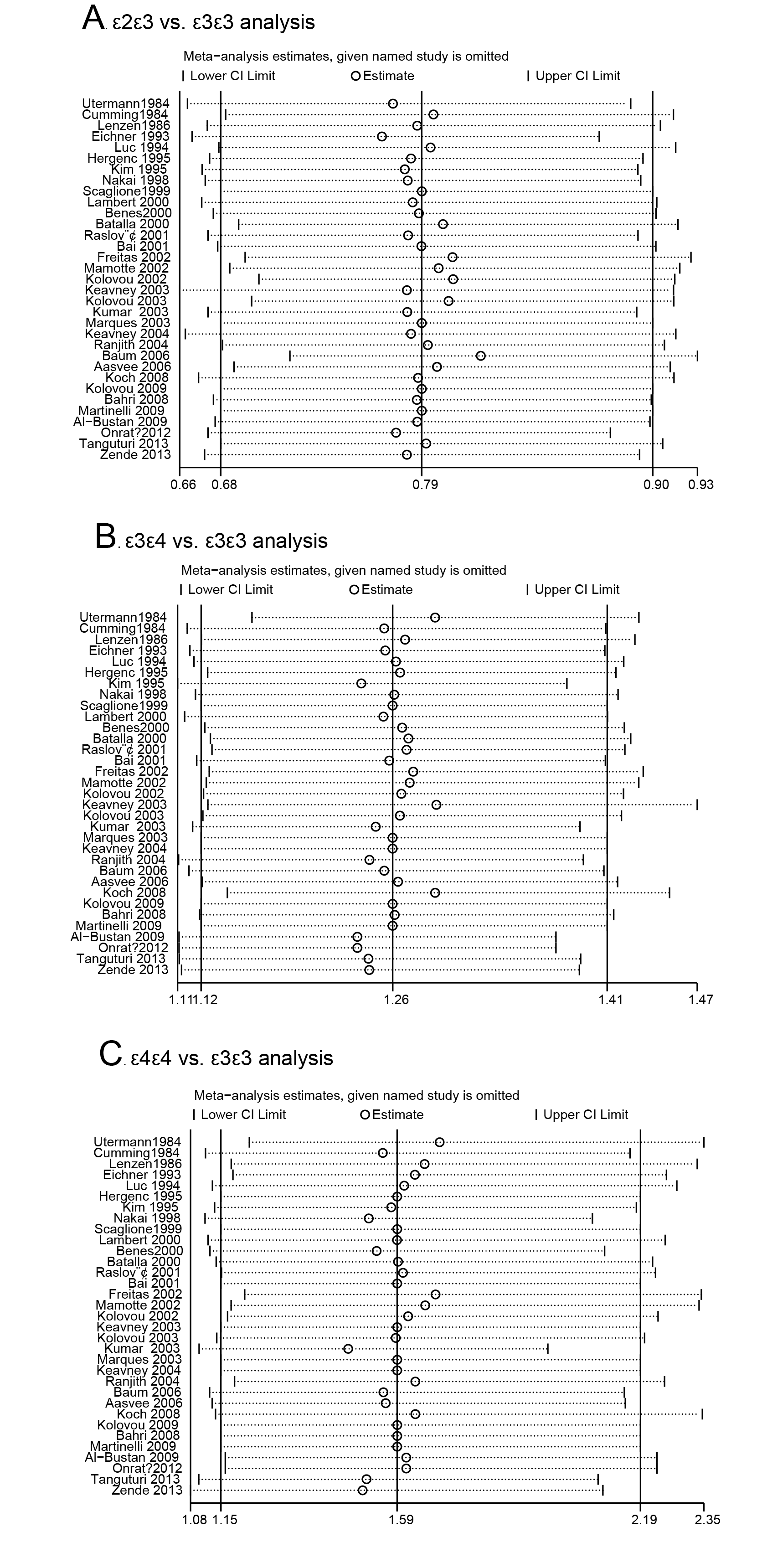

Supplement: Figure S7 — Influence analysis of ApoE gene polymorphism and MI risk: A) ε2ε3 vs. ε3ε3 analysis; B) ε3ε4 vs. ε3ε3 analysi; C) ε4ε4 vs. ε3ε3 analysis. (TIF) [file pone.0104608.s007.tif]
